# Microvibration Testing and Decoupling for Space Payloads with Large Inertia, High Stiffness, and Discrete Interfaces

**DOI:** 10.3390/s25237352

**Published:** 2025-12-03

**Authors:** Renkui Jiang, Wei Liang, Libin Wang, Haibing Su, Yanqing Zhang, Tonglei Jiang, Junfeng Du, Ang Zhang

**Affiliations:** 1 Institute of Optics and Electronics, Chinese Academy of Sciences, Chengdu 610209, China; jiangrenkui@ioe.ac.cn (R.J.); liangwei@ioe.ac.cn (W.L.);; 2 National Time Service Center, Chinese Academy of Sciences, Xi’an 710600, China

**Keywords:** microvibration testing, coupled microvibration analysis, semi-physical simulation, China Space Station Telescope

## Abstract

As the core observation instrument of the China Space Station Telescope (CSST), the Survey Camera (SC) generates microvibrations that significantly degrade the telescope’s imaging quality. Consequently, evaluating the microvibration response of the SC is of critical importance. However, for large-inertia, high-stiffness payloads like the SC with discrete interfaces, structural coupling between the payload and the test system leads to distortions in microvibration test results. Since the vibration transmission under structural coupling is not a simple series superposition, and the transfer functions of each link in the transmission path as well as the coupling correction matrices are difficult to obtain, this paper proposes a semi-physical simulation method for microvibration decoupling. The method first establishes a coupled finite element model of the SC and the test system. The model is iteratively modified based on the results of modal tests and transmissibility tests to ensure consistency with the dynamic characteristics of the actual coupled system. The model is validated through microvibration response tests, and the results show good agreement between the model and the actual system (the RMS deviation of force/torque is less than 5%). After stripping the test system from the modified coupled model, the intrinsic microvibration responses of the SC can be extracted, achieving the dynamic decoupling analysis of the complex coupled system.

## 1. Introduction

Against the backdrop of continuous breakthroughs in deep space exploration technology, performance requirements for spacecraft in terms of resolution, stability, and pointing accuracy are becoming increasingly stringent [1,2,3]. The impact of disturbances such as vibrations on such spacecraft is particularly significant [4,5], such as the Hubble Space Telescope (HST) [6] and the James Webb Space Telescope (JWST) [7,8]. Even if only a 1 N microvibration load is applied to the optical payloads of spacecraft, it will cause severe degradation of imaging quality, reaching an unacceptable level. As a unique mechanical phenomenon in the microgravity environment, microvibration has implications far beyond the literal meaning of “low-level disturbance”. This type of vibration spans a wide frequency range, extending from near-static sub-hertz levels up to kilohertz levels, covering the critical operating frequency bands of various sensitive payloads on spacecraft [9,10]. Its sources are complex and diverse, such as the pulsed operation of attitude control engines, the high-speed rotation of reaction wheels, the reciprocating motion of refrigeration compressors, and the thermally induced jitter of solar panels [11,12,13]. Due to the obvious differences in vibration characteristics among different vibration sources, during the microvibration suppression process, it is necessary to carry out adaptive suppression designs based on specific vibration source characteristics to effectively reduce the accumulation and amplification effects of vibrations [14,15]. Thus, mastering the vibration responses of the vibration sources is particularly important.

**Figure 1:**
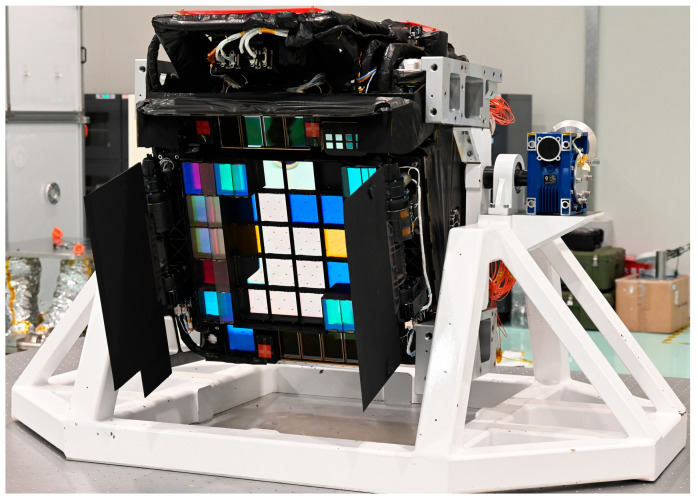
The fully integrated Survey Camera.

As China’s largest and most sophisticated next-generation space-based astronomical telescope, the China Space Station Telescope (CSST) features a 2-m primary mirror aperture [16,17,18], with a survey field of view of 1.1 × 1.0 square degrees and an angular resolution close to that of the HST. The radius encircling 80% energy of the point spread function is required to be no more than 0.15″ [19,20], including all wavefront errors and dynamical effects (microvibration). The Survey Camera (SC, as shown in Figure 1), a core observational instrument aboard the CSST [21], is assigned 70% of the total observational tasks. Its design prioritizes high-resolution, large-scale multiband imaging capabilities, with a primary focal plane size of 500 × 600 mm and equipped with 3.3 billion pixels. The SC is expected to exceed GAIA [22,23] and become the space astronomical camera with the highest number of pixels to date. Inside the SC, the Cryocooler Assembly (CCA) serves as an inherent vibration source. This assembly operates continuously during the camera’s imaging process, and its operational vibrations are transmitted to the CSST through paths such as the camera’s support structure and heat pipes, inducing jitter of the telescope’s optical axis [24]. This results in displacement of the observed target relative to the detector during exposure, thereby significantly degrading the spatial resolution of the images. Therefore, it is particularly critical to gain an in-depth understanding of the microvibration response characteristics of the SC and to conduct adaptive design of the telescope based on its response laws to weaken the transmission and amplification effects of microvibrations.

Currently, the prevalent method for evaluating microvibration responses of space payloads is to conduct microvibration tests on them under blocked or infinite-impedance configurations, where force sensors are used to quantify microvibration responses at the interface [25]. However, for test objects such as SC, which feature large inertia, high stiffness, and discrete interfaces, it is difficult for the test system to ensure that the test object meets the blocked or infinite-impedance installation conditions. This leads to structural coupling between the two after installation, thereby causing the test results to fail to truly reflect the microvibration response of the test object. The core issue lies in how to extract the true microvibration response of the test object from the microvibration test results when there is a high degree of structural coupling between the test system and the test object.

In the field of coupled microvibration research, reference [26] characterize the structural coupling effect between the flywheel and the spacecraft mounting surface by introducing a force filter. This force filter mainly consists of three components: first, the spacecraft transfer function obtained through finite element analyses; second, the point dynamic mass at the mounting points of the flywheel assembly tested under static conditions; third, the microvibration response of the flywheel assembly under blocked or infinite-impedance installation conditions. Similar studies have been conducted in references [27], which incorporate the gyroscopic effect generated during the rotation of the flywheel. It is worth noting that the reverse application of this method can achieve the decoupling of microvibration tests for vibration sources—specifically, starting from the tested microvibration response under the coupled state, it can back-calculate the microvibration response of the vibration source under blocked or infinite-impedance installation conditions. However, this method has certain limitations and is only applicable to scenarios where the stiffness of the test system is significantly higher than that of the test object, and the mounting interface between the two is in a clear and single series connection form.

Another commonly used approach is the substructuring method, which decomposes the global structure into independent substructures and achieves dynamic analysis by satisfying interface compatibility and equilibrium conditions [28]. Typical implementations such as component mode synthesis rely on clear substructure partitioning and accurate acquisition of interface dynamic parameters. However, for the SC, there are multiple mounting interfaces with large spans between them, resulting in a lack of distinct installation boundaries between the test object and the test system, as well as significant multi-path coupling effects in vibration transmission. These characteristics make the partitioning of substructures and the validation of interface condition assumptions extremely challenging, thus greatly restricting the direct application of the substructuring method.

In addressing structural coupling issues during spacecraft vibration testing, virtual testing has emerged as a comprehensive framework that models the full interaction between the test facility, specimen, and control system, encompassing nonlinear and transient effects [29]. This technique has been extensively validated by the European Space Agency (ESA) for complex spacecraft testing scenarios. Notably, ESA developed virtual testing platforms for its QUAD and HYDRA shakers in the late 2010s, successfully applying them to missions such as BepiColombo (a joint ESA/JAXA Mercury exploration mission) and tests with the TEDY mass dummy spacecraft [30]. The HYDRA 6-axis shaker, capable of simultaneous multi-degree-of-freedom transient testing, was integrated into a virtual model that included MTS control systems and high-fidelity finite element models of test specimens. This integration revealed that structural coupling between the shaker and spacecraft could alter fundamental modal frequencies by over 20% and affect more than 39% of resonance modes, highlighting the criticality of full-system modeling [30]. Virtual testing not only improved post-test correlation accuracy (with FRAC values increasing by an average of 0.2) but also enabled reliable pre-test prediction of unexpected dynamic behaviors, overcoming the limitations of traditional finite element analysis that neglects facility-induced uncertainties [30]. Building on the full-system dynamic modeling philosophy of virtual testing, this paper proposes a targeted microvibration testing and decoupling method for large-inertia, high-stiffness payloads with discrete interfaces (e.g., the Survey Camera, SC). It introduces a closed-loop decoupling process consisting of “model establishment—model validation—test system stripping—intrinsic response extraction”, which efficiently separates the intrinsic microvibration characteristics of the test object from the coupling interference of the test system. This thus provides an engineering-feasible solution for microvibration testing of similar large-inertia, high-stiffness payloads with discrete interfaces.

In view of payloads such as the SC, which feature large inertia, high stiffness, as well as multiple and discrete mounting interfaces, this paper proposes a semi-physical simulation method for their microvibration testing and decoupling. In this paper, Section 2 describes the vibration sources and transmission path of the SC and the composition of Microvibration Testing System. Section 3 establishes coupled dynamic models for the SC and MVTS and identifies challenges in decoupling due to multiple paths and dynamic mass acquisition. Section 4 proposes a semi-physical simulation method. Section 5 describes in detail the testing of the SC and the decoupling process of its microvibration responses.

## 2. Structural Coupling Between the Survey Camera and Microvibration Testing System

**Figure 2:**
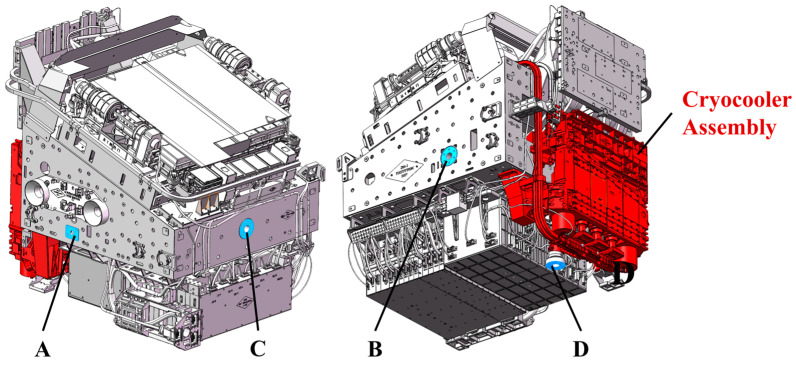
The mounting interface between the SC and CSST.

All individual units of the SC are integrated into a support structure fabricated from M55J carbon fiber composite material. This support structure adopts a high-stiffness design, and its fundamental frequency exceeds 100 Hz when fully loaded with fixed mounting interfaces. As the vibration source of the SC, the Cryocooler Assembly (CCA) internally integrates four pulse tube cryocoolers (PTCs), among which three are used to cool the focal plane array to below 185 K, and one is utilized to cool the short-wave infrared detector to below 80 K. Each group of cryocoolers is equipped with a compressor. During the operation of the cryocoolers, the piston inside the compressor reciprocates to drive the working fluid to flow, thereby achieving refrigeration. The operating frequency of the compressor can be adjusted within the range of 75–85 Hz, with additional vibrations occurring at its 2nd and 3rd harmonics. To reduce the vibration of the CCA, vibration isolators are installed between the CCA and the support structure of the SC. After vibration isolation, the natural frequency of the CCA is about 10 Hz. Vibrations generated by the CCA are transmitted to the SC through the vibration isolators and heat pipes, and then transferred from the SC to the CSST via the mounting interfaces. The mounting interfaces of the SC are illustrated in Figure 2, with four interfaces (A, B, C, and D) distributed in four directions. In the shutter-closed state, the SC has dimensions of 895 mm × 1047 mm × 1047 mm and a launch mass of 450 kg.

Microvibrations transmitted from the SC to the CSST can cause jitter in the optical axis of the telescope. The Fast Steering Mirror (FSM) configured in the optical system of CSST possesses precise image stabilization capabilities, which can effectively compensate for the impact of low-frequency vibrations below 8 Hz on imaging. However, for the typical 75–80 Hz vibrations generated by the cryocooler, the dynamic response capability of the FSM is limited, making it difficult to achieve complete suppression. The residual optical axis jitter after compensation by the FSM will cause the observed target to displace relative to the detector during exposure, thereby significantly reducing the spatial resolution of the image. According to the decomposed requirements of the telescope specifications, within the frequency range of 8–300 Hz, the root mean square (RMS) values of the vibration force and vibration torque transmitted from the SC to the CSST must be controlled to ≤0.4 N and ≤0.1 N·m, respectively.

**Figure 3:**
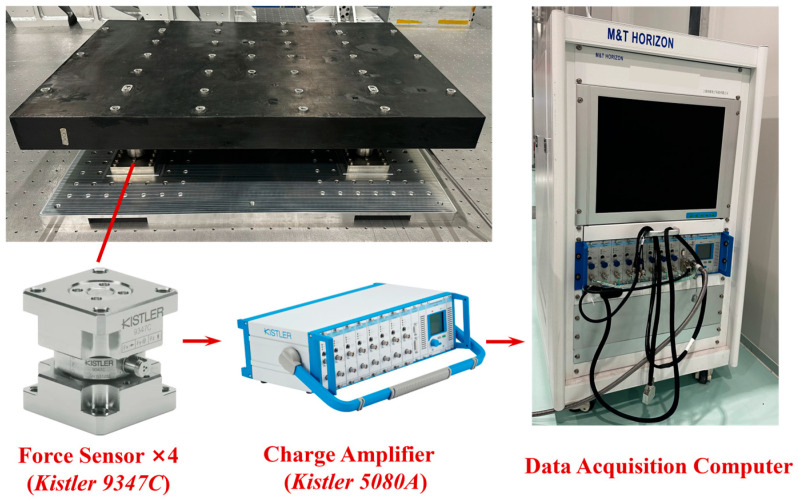
Microvibration Testing System.

To verify whether the microvibration response of the SC meets the requirements, a microvibration test was conducted. The microvibration test system (MVTS) employed in the test is illustrated in Figure 3. This system integrates four Kistler 9347C force sensors—IEPE-type triaxial sensors with sensitivities of −8 pC/N (for Fx and Fy directions) and −3 pC/N (for Fz direction)—which are symmetrically mounted between the foundation and the carbon fiber mounting baseplate. The total test force and torque were derived from the measured forces of the four sensors and the distances between them.

The force sensors convert mechanical forces into electrical signals, which are then amplified by a charge amplifier (Kistler 5080A; measuring range: ±2~±2,200,000 pC; measurement uncertainty: 0.3%) and transmitted to a data acquisition computer for processing. The MVTS enables high-precision measurements with a force measuring range of 600 N and a torque measuring range of 12 N·m, achieving a force resolution of 0.005 N and a torque resolution of 0.001 N·m.

**Figure 4:**
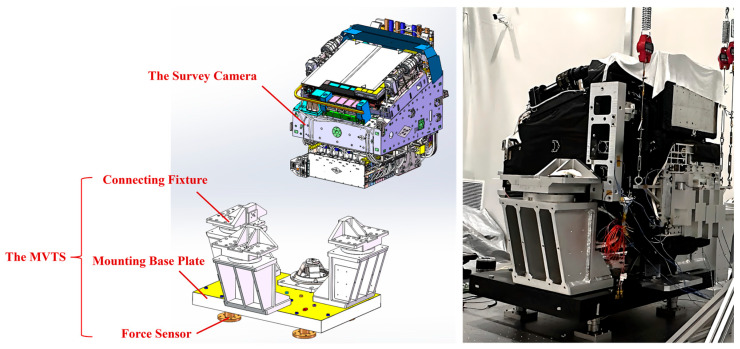
The SC-MVTS coupled system.

The SC is connected to the mounting base plate via a dedicated connecting fixture, as shown in Figure 4.

**Figure 5:**
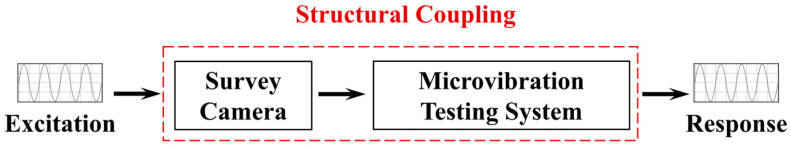
Vibration transmission path of the SC-MVTS coupled system.

The vibration transmission path after the SC is connected to the MVTS is shown in Figure 5. Due to the SC itself having characteristics of large inertia and high stiffness, and its mechanical mounting interfaces adopting a distributed design, structural coupling is formed between the SC and the MVTS after they are connected. This coupling leads to differences in dynamic characteristics between the SC-MVTS coupled system and the standalone SC, thereby making the tested microvibration response unable to truly reflect the microvibration response of the SC itself.

**Figure 6:**
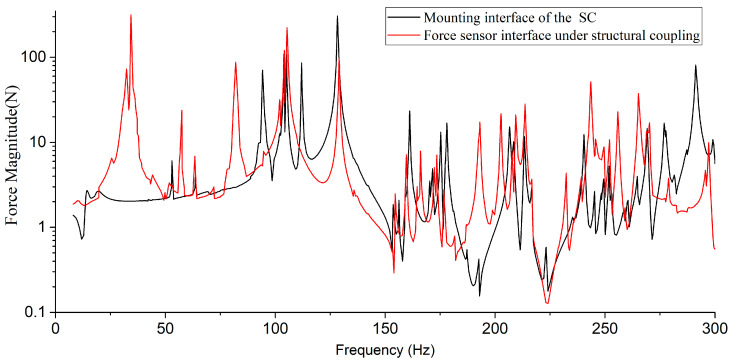
Vibration transmissibility curves of the SC and the SC-MVTS coupled system.

To verify the differences in dynamic characteristics between the SC itself and the SC-MVTS coupled system, finite element models (FEMs) of both were established, and their respective vibration transmissibility curves were analyzed. In Figure 6, the blue curve represents the vibration transmissibility curve from the CCA mounting interface to the SC mounting interface (where the four mounting interfaces A, B, C, and D are connected via rigid elements, with the independent point of the rigid elements set at the centroid of the SC). The red curve represents the vibration transmissibility curve from the CCA mounting interface to the force sensor mounting interface of the MVTS. The two curves show significant differences: the stiffness of the coupled system is significantly lower than that of the SC itself, and multiple resonance peaks appear within the excitation frequency range of the CCA, which will lead to an overestimation of the tested microvibration response.

## 3. Coupled Microvibration Analysis Model

To eliminate the influence of the MVTS on the micro—vibration testing of the SC, a Coupled Microvibration Analysis Model is established to analyze the coupling principle. Under the excitation of the CCA, the microvibration force and torque responses of the SC can be expressed as:

(1) $$F_{SC}\left(s\right)=G_{S1}F_{CCA}\left(s\right)$$

where $F_{CCA}$ is the disturbance force and torque of the CCA, and is a known quantity, and $F_{SC}$ is the microvibration response of the SC; the resultant force and resultant torque of all interfaces constitute the microvibration force and torque responses of the SC. $G_{S1}$ is the transfer function of the SC relating $F_{CCA}$ to $F_{SC}$. The key to the problem lies in obtaining $G_{S1}$. Due to the dispersed interfaces of the SC, the excitation from the CCA will be transmitted to each interface through different paths, making the solution of $G_{S1}$ challenging.

For a flexible body with a limited volume, discretized into a finite number of degrees of freedom (DOF), and subjected to external forces and torques, the corresponding equation of motion (EOM) is:

(2) $$M\ddot{x}+C\dot{x}+Kx=F$$

In the equation, *M* is the mass matrix, *C* is the damping matrix, *K* is the stiffness matrix, *x* is the vector of displacements and rotations, and *F* is the vector of forces and torques.

By performing the Laplace transform on Equation (1) and assuming the initial condition of *x*(*t*) is zero, we obtain:

(3) $$M\ddot{X}\left(s\right)+C\ddot{X}\left(s\right)s^{-1}+K\ddot{X}\left(s\right)s^{-2}=F\left(s\right)$$

which can be written in the following form:

(4) $$F\left(s\right)=G\ddot{X}\left(s\right)$$

The transfer function matrix G, which links the applied loads F to the accelerations $\ddot{X}$, is defined as follows:

(5) $$G=M+Cs^{-1}+Ks^{-2}$$

G is a 6 × 6 matrix with all elements dependent on frequency. This matrix stands for the “driving-point” dynamic mass of the flexible body, which characterizes the relationship between the load force applied at this point and the acceleration.

**Figure 7:**
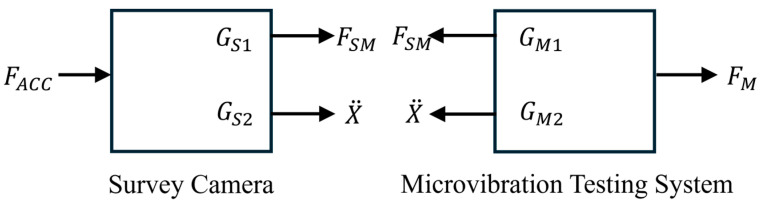
Schematic diagram of coupling between the SC and the MVTS.

Figure 7 shows the schematic diagram of coupling between the SC and the MVTS. The SC equation of motion (EOM) is obtained as:

(6) $$F_{SM}\left(s\right)={R_{S}G_{S1}F}_{CCA}\left(s\right)+G_{S2}\ddot{X}\left(s\right)$$

where $\ddot{X}$ is linear and angular accelerations at the SC-MVTS mounting interface, $F_{SM}$ is the reaction forces and torques at the SC-MVTS mounting interface, $G_{S2}$ is the driving-point dynamic mass of the SC at the SC-MVTS mounting interface. Due to the comparable inertia and stiffness between the SC and the MVTS, $G_{S1}$ has changed after connection and coupling. Here, $R_{S}$ is introduced to correct the transfer functions of the SC, and it is a 6 × 6 matrix related to frequency.

The MVTS’s equation of motion (EOM) is obtained as:

(7) $$F_{M}\left(s\right)={{R_{M}G}_{M1}F}_{SM}\left(s\right)$$

(8) $$F_{SM}\left(s\right)=G_{M2}\ddot{X}\left(s\right)$$

where $F_{M}$ is the vibration forces and torques tested by the MVTS after the system is coupled, $G_{M1}$ is the transfer function of the MVTS relating $F_{SM}$ to $F_{M}$, $R_{M}$ is the transfer function correction matrix of the MVTS. $G_{M2}$ is the driving-point dynamic mass of the MVTS at the SC-MVTS mounting interface.

By combining Equations (1) and (6)–(8), the microvibration force and torque responses of the SC are obtained:

(9) $$F_{SC}\left(s\right)=\left(G_{M2}-G_{S2}\right){\left({R_{S}R}_{M}G_{M1}G_{M2}\right)}^{-1}F_{M}\left(s\right)$$

Given that the SC and the MVTS do not feature an independent series-mounted interface, with multiple coupling paths involved, the dynamic masses $G_{S2}$ and $G_{M2}$ at the interface are difficult to obtain. Additionally, the transfer function correction matrix $R_{S}$ and $R_{M}$ are also difficult to obtain through testing.

## 4. Principle of Microvibration Decoupling in Semi-Physical Simulation

**Figure 8:**
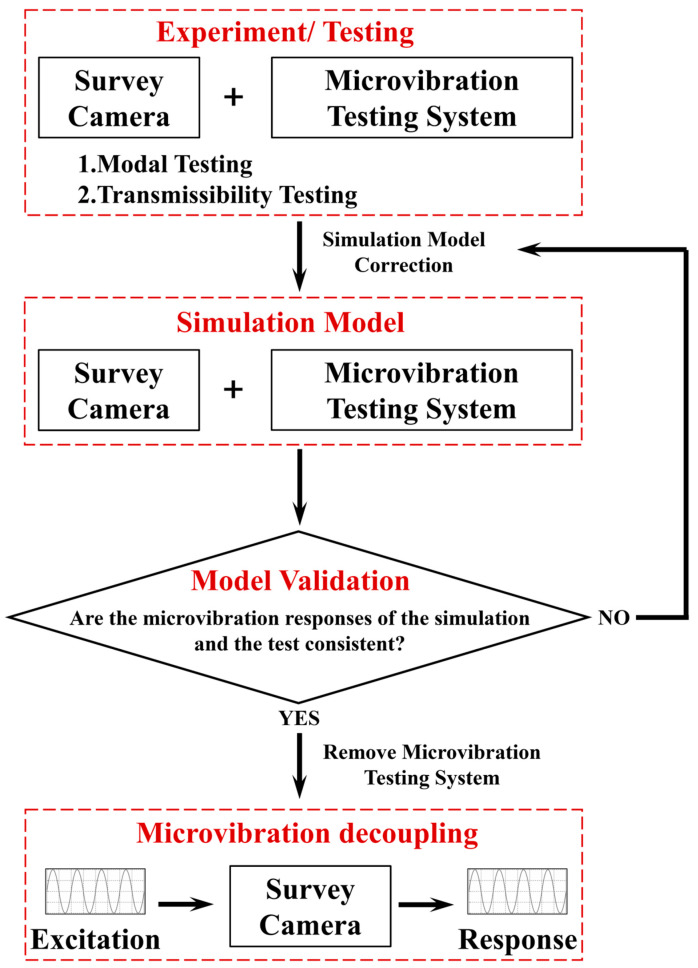
Schematic diagram of the semi-physical simulation method.

Analysis of the coupled microvibration model reveals significant difficulties in directly obtaining $G_{S1}$ through tests and theoretical derivations. To address this issue, a semi-physical simulation method is proposed (schematic diagram shown in Figure 8), with the specific process as follows:

First, the SC is mounted on the MVTS to construct the SC-MVTS coupled system. Subsequently, modal testing is carried out to identify the inherent vibration characteristics of this coupled system, and meanwhile, transmissibility testing is performed to quantify the vibration transmission laws.

Subsequently, an FEM of the SC-MVTS coupled system is constructed. Based on the test results of modal testing and transmissibility testing, iterative corrections are performed on the model.

Then, to verify the accuracy of the simulation model, microvibration response testing is conducted on the SC-MVTS coupled system. This test can characterize the entire transmission chain from the CCA, through the SC and MVTS, to the force sensors, and is directly related to the force and torque physical quantities of research interest. By comparing the results of the microvibration response testing and simulation analysis, subsequent steps can be carried out only after the two achieve good consistency; otherwise, the model will be further revised.

Once the model is highly consistent with the dynamic characteristics of the actual system, the MVTS is “stripped” from the coupled model, while the FEM of the SC itself is retained. This model fully inherits the corrected dynamic characteristics of the SC, including connection stiffness, damping properties, and modal parameters. Subsequently, the CCA excitation is input into the model, and the micro-vibration response of the SC is calculated independently. This method overcomes the limitations of decoupling analysis on the dynamic behavior of the target object in complex coupled environments, enabling decoupling from “coupled system response” to “intrinsic characteristics of the target object”.

## 5. Microvibration Testing and Decoupling

### 5.1. Testing and Simulation Model Correction

**Figure 9:**
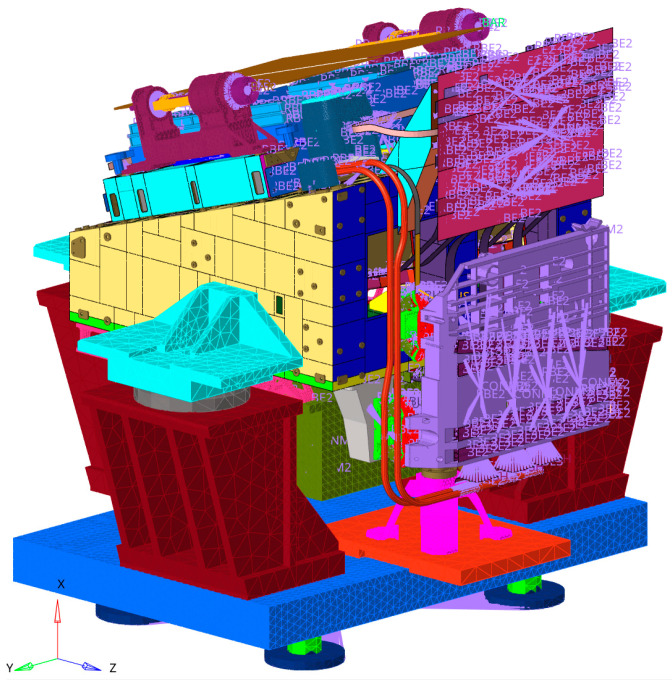
FEM of the SC-MVTS coupled system.

To ensure the modeling accuracy of the system, a hybrid modeling approach using solid, shell, beam, and mass point elements was adopted. The FEM of the SC-MVTS coupled system contains over 2 million elements, as shown in Figure 9.

First, modal tests were conducted on the SC-MVTS coupled system. The tested modal frequencies and mode shapes are listed in Table 1. Based on the test data, the connection stiffness at each interface of the model and the material property parameters of some structures were adjusted to correct the natural frequencies of the model. After modification, the deviation between the simulated and tested frequencies was controlled within 5.6%, and the mode shapes of each order remained consistent, achieving alignment between the simulation model and the test in both frequency points and mode shapes.

**Figure 10:**
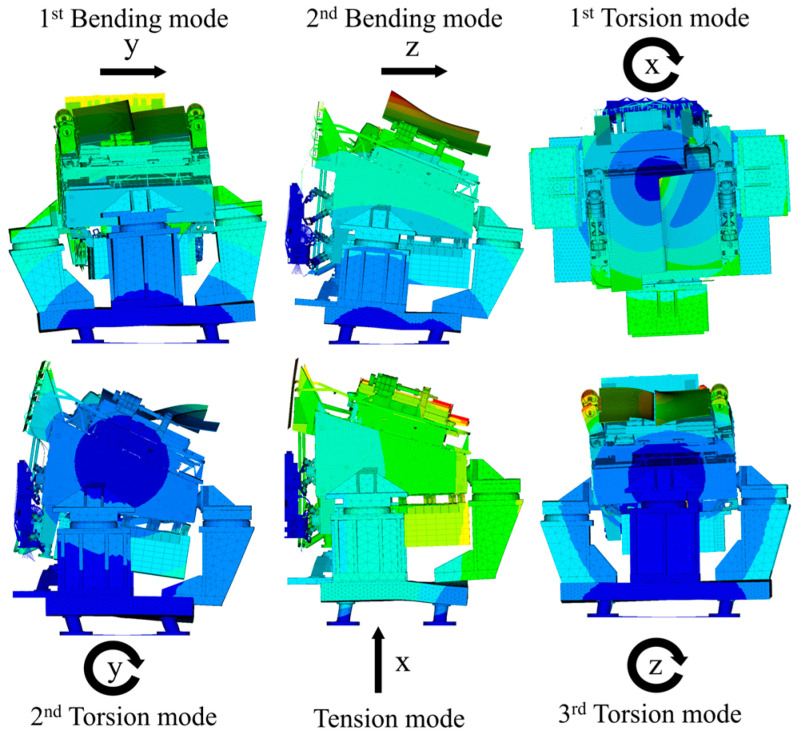
Mode shapes of the SC-MVTS coupled system.

The mode shapes of the coupled system are shown in Figure 10. The 1st to 6th mode shapes correspond to: y-direction bending mode, z-direction bending mode, x-direction torsion mode, y-direction torsion mode, x-direction tension mode, and z-direction torsion mode, respectively.

**Figure 11:**
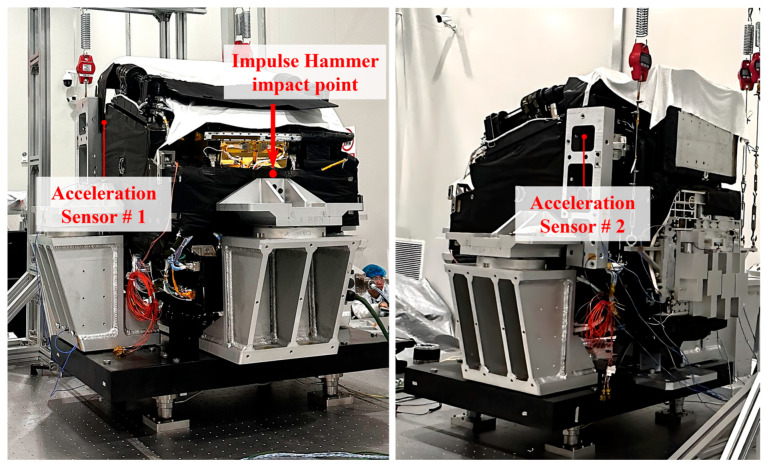
Arrangement of impulse hammer impact point and acceleration sensors.

On this basis, testing was conducted to obtain the acceleration/force transmissibility curves of the SC-MVTS coupled system. An impulse hammer was used to impact different positions on the coupled system, and the acceleration responses at specific characteristic locations of the system were tested. Through calculation, the acceleration/force transmissibility curves between the strike points and test points of the coupled system were derived. Based on these transmissibility curves, the damping values at various frequencies in the simulation model were corrected to make its vibration transmission characteristics consistent with the tested data, thereby improving the model accuracy. As shown in Figure 11, the layout of one set of impact and test positions is as follows: the impact point of the impulse hammer is located on the connecting fixture, while the acceleration test points are arranged on both sides of the support structure.

**Figure 12:**
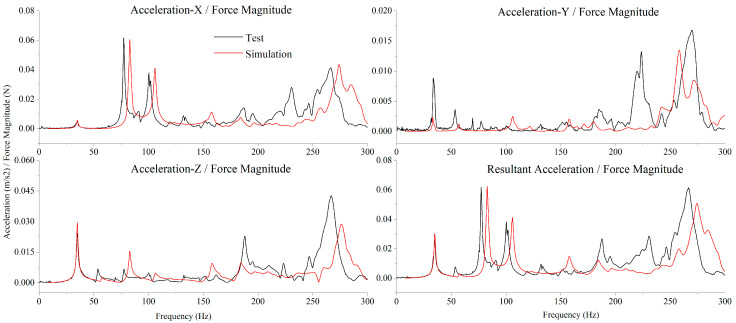
Transmissibility curves of acceleration/force at characteristic point #1.

**Figure 13:**
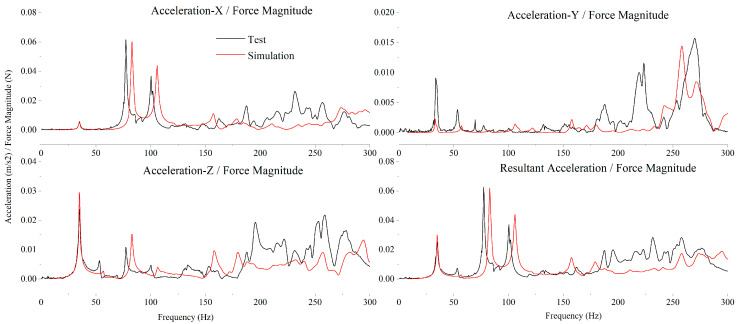
Transmissibility curves of acceleration/force at characteristic point #2.

After model correction, the test acceleration/force transmissibility curves at various positions showed good consistency with the simulation results. As presented in Figure 12 and Figure 13, the acceleration/force transmissibility curves of the above two test points demonstrated fine agreement between the test results (black curves) and simulation results (red curves) in multiple dimensions: the positions of the main resonance peaks were generally consistent, the vibration magnitudes in all directions matched well, and the amplitude variation trends were synchronized. Over the wide frequency range, both the distribution pattern of resonance peaks and the attenuation law in non-resonant regions showed identical characteristics. Although slight differences existed in the response details of local frequency ranges and the amplitudes of some resonance peaks, the model accurately captured core vibration characteristics such as the main resonance frequencies and mode shape excitation response distributions, thereby fully validating the simulation accuracy of the modified SC-MVTS coupled system model.

### 5.2. Model Validation

**Figure 14:**
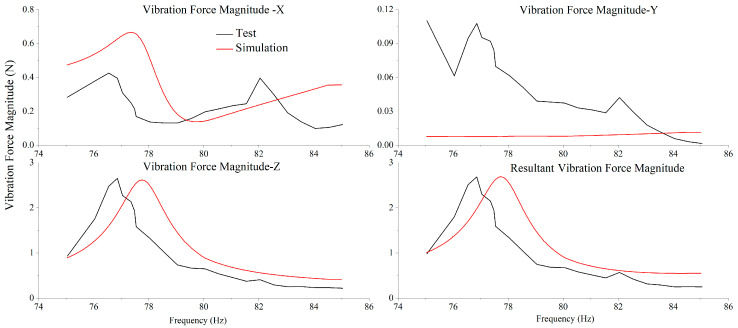
Tested and simulated vibration force responses.

To verify the accuracy of the model, microvibration response testing was conducted on the SC-MVTS coupled system under the operating condition of the CCA, and the test results were compared with the simulation results of the modified model. In Figure 14, the black curve represents the tested vibration force response when the CCA operates within the frequency range of 75–85 Hz; the red curve denotes the microvibration response simulated at the force sensor interface after inputting the cryocooler excitation into the modified simulation model. The two curves show good agreement, with comparable vibration response magnitudes in all directions. The RMS values of the resultant vibration force from the test and simulation are 4.46 N and 4.60 N, respectively, with a deviation of only 3%.

**Figure 15:**
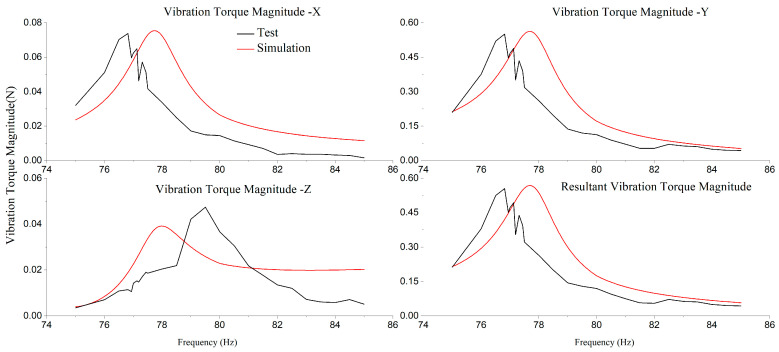
Tested and simulated vibration torque responses.

The response curves of the vibration torque from the test and simulation also show high consistency, as presented in Figure 15. The RMS values of the resultant torque for both are 1.05 N·m and 0.98 N·m, respectively, with a deviation of 5%. These results indicate that the simulation model is highly consistent with the actual coupling system.

### 5.3. Microvibration Decoupling

**Figure 16:**
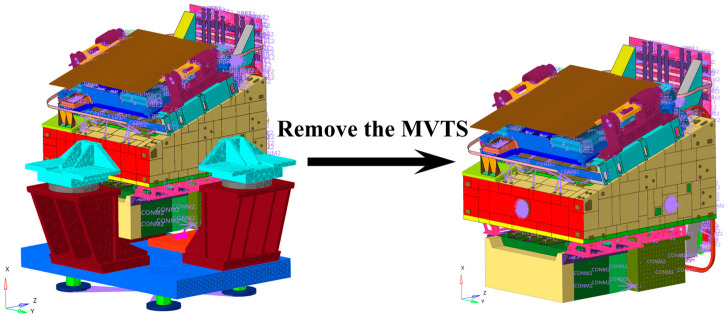
Remove the MVTS from the coupled model.

After modification and validation, the FEM of the SC-MVTS coupled system has achieved high simulation accuracy. On this basis, the MVTS was removed from the model, while the FEM of the SC itself was retained. This model fully inherits the corrected dynamic properties of the camera’s internal components, including connection stiffness, damping characteristics, and modal parameters, as shown in Figure 16.

**Figure 17:**
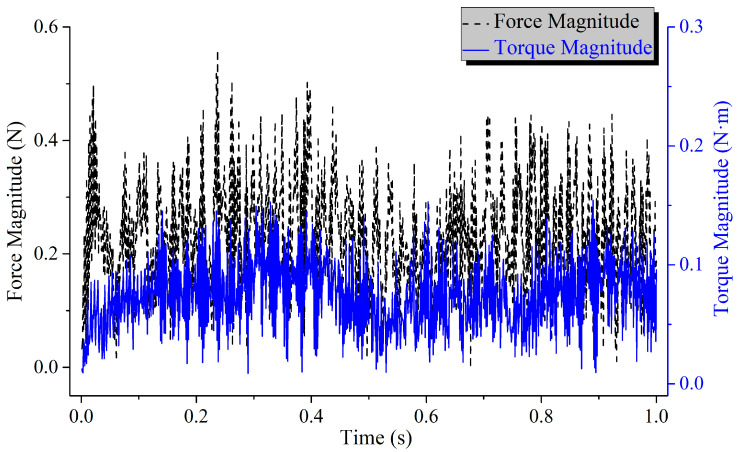
Microvibration time domain response of the SC.

**Figure 18:**
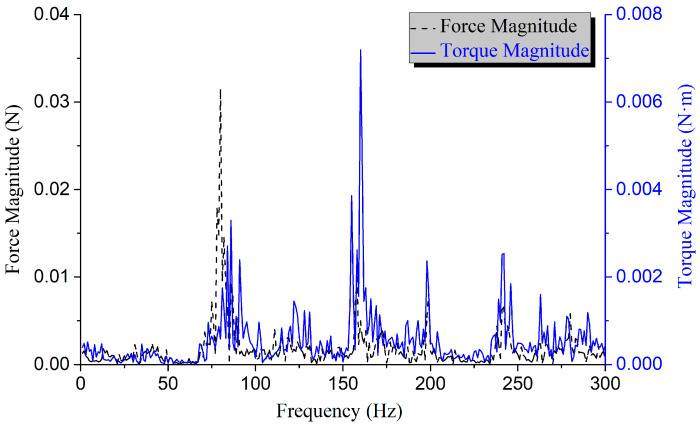
Microvibration frequency domain response of the SC.

The CCA excitation was applied to the FEM of the SC, and simulation generated the response curves of microvibration forces and torques at the mounting interface of the SC (as shown in Figure 17 and Figure 18). Calculations indicate that within the frequency range of 8–300 Hz, the RMS values of the vibration force and torque are 0.25 N and 0.08 N·m, respectively. Both meet the microvibration requirements of the SC, i.e., vibration force ≤ 0.4 N and torque ≤ 0.1 N·m. In addition, the SC has been delivered and successfully integrated with the CSST. Subsequent relevant optical imaging tests have been carried out, and the imaging quality met the expectations.

## 6. Conclusions

For payloads such as the Survey Camera (SC), which feature large inertia, high stiffness, and discrete mounting interfaces, structural coupling tends to occur between the payload and the testing system during microvibration testing, thereby leading to distortions in the test results. Since the vibration transmission in a coupled system is not a simple series superposition, the method of calculating the transfer functions of each link in the transmission path and the coupling correction matrix faces the problem that each component is difficult to obtain, making it hard to achieve microvibration decoupling.

To address this challenge, this paper proposes a semi-physical simulation method to solve the problem of microvibration test decoupling for the SC. Firstly, a coupled system of the Survey Camera-Microvibration Test System (SC-MVTS) is built, and at the same time, the FEM of the SC-MVTS coupled system is constructed. Modal tests and transmissibility tests are conducted on the SC-MVTS coupled system, and the model is iteratively modified based on the test data to make the simulation results as close as possible to the test data, ensuring that the dynamic characteristics of the SC-MVTS coupled system simulation model are consistent with those of the actual system.

Subsequently, the coupled model is validated through microvibration response tests, and the results demonstrate that the simulation results of the modified model are in good agreement with the tested results, and the deviations of the RMS values of the resultant force and torque of the vibration response are within 5%. Then, the MVTS is stripped from the SC-MVTS coupled model to separate the inherent dynamic characteristics of the SC. Based on the excitation of the CCA, the microvibration response of the SC can be calculated, achieving the dynamic decoupling analysis of the complex coupled system. Decoupling analysis indicates that in the frequency range of 8–300 Hz, the RMS values of the microvibration force and torque of the SC are 0.25 N and 0.08 N·m, respectively, meeting the requirements of the SC (≤0.4 N and ≤0.1 N·m).

Future work can be extended in several directions to enhance the practical application and generalization ability of the proposed method. First, the methodology can be adapted to other types of space payloads with diverse structural characteristics (e.g., flexible attachments, multi-source excitation, or irregular interface distributions), expanding its engineering application scope to satellite antennas, precision spectrometers, and other high-precision space instruments. Second, integrating machine learning algorithms (such as neural networks or Gaussian process regression) into the finite element method (FEM) iterative correction process may improve the accuracy and efficiency of the model, especially for complex coupled systems with high-dimensional parameters. Third, formulating a standardized operating procedure for the proposed decoupling method will facilitate its application in industrial testing scenarios, reduce reliance on expert experience, and improve test repeatability. These extensions are expected to provide more comprehensive solutions for the microvibration evaluation and control of high-precision space systems, supporting the development of next-generation deep space exploration missions.

## Figures and Tables

**Table 1:** Modal frequencies of the SC-MVTS coupled system.

Modal Order	Tested (Hz)	Simulated (Hz)	Deviation	Mode Shape
1st	33.0	33.0	0.0%	1st Bending
2nd	34.1	34.8	2.0%	2nd Bending
3rd	53.8	57.0	5.6%	1st Torsion
4th	77.2	80.6	4.5%	2nd Torsion
5th	101.3	105.7	4.2%	Tension
6th	130.9	123.6	-5.6%	3rd Torsion

## Data Availability

The data presented in this study is available on request from the corresponding author.

## Abbreviations

The following abbreviations are used in this manuscript:

CCA	Cryocooler Assembly
CSST	China Space Station Telescope
DOF	Degrees of Freedom
EOM	Equation of Motion
ESA	European Space Agency
FEM	Finite Element Model
FSM	Fast Steering Mirror
FRAC	Frequency Response Assurance Criterion
HST	Hubble Space Telescope
IEPE	Integrated Electronics Piezoelectric
JAXA	Japan Aerospace Exploration Agency
JWST	James Webb Space Telescope
MVTS	Microvibration Testing System
PTC	Pulse Tube Cryocooler
RMS	Root Mean Square
SC	Survey Camera
